# Infections following the application of leeches: two case reports and review of the literature

**DOI:** 10.1186/1752-1947-6-364

**Published:** 2012-10-25

**Authors:** Benjamin Maetz, Ralph Abbou, Jean Baptiste Andreoletti, Catherine Bruant-Rodier

**Affiliations:** 1 Department of Plastic and Maxillo-facial Surgery, Hôpitaux Universitaires de Strasbourg, 1, Place de l’Hôpital, Strasbourg Cedex, 67091, France; 2 Department of Plastic and Reconstructive Surgery, Hospital Emile Muller, 20, Rue Laennec, Mulhouse, 68100, France

**Keywords:** *Aeromonas veronii* biovar sobria, Infection, Leeches, TRAM flap, Venous congestion

## Abstract

**Introduction:**

Since the 1980s, leeches have been ingeniously used in the management of venous flap congestion. The presence of anticoagulative substances in their saliva improves the blood drainage. Their digestive tract contains several bacterial species, the main ones being *Aeromonas hydrophila* and *Aeromonas veronii* biovar sobria, which contribute to the digestion of ingested blood. These bacteria can be the cause of infections.

**Case presentation:**

We report two cases of septicemia related to *Aeromonas veronii* biovar sobria that presented after leeches had been applied to congested transverse rectus abdominis myocutaneous flaps for delayed mammary reconstructions.

Patient number 1 was a 55-year-old Caucasian woman who underwent a delayed breast reconstruction procedure. On the sixth postoperative day she showed a clinical presentation of septicemia. *Aeromonas veronii* biovar sobria was identified in the patient’s skin and blood bacteriological samples. Her fever ceased after 4 days of antibiotic treatment.

Patient number 2 was a 56-year-old Caucasian woman who underwent a delayed breast reconstruction procedure. On the seventh postoperative day we noticed that she showed a clinical presentation of septicemia. *Aeromonas veronii* biovar sobria was identified in the patient’s blood cultures and local bacteriological samples. An antibiogram showed resistance to amoxicillin/clavulanic acid. Her fever ceased on the eleventh postoperative day after 4 days of antibiotic treatment.

**Conclusion:**

The rate of infection after application of leeches is not negligible. The concentration of *Aeromonas* inside the digestive tracts of leeches largely decreases when the patient is under antibiotic therapy. These germs are sensitive to third-generation cephalosporins and fluoroquinolones and resistant to amoxicillin/clavulanic acid. We recommend preventive treatment based on classical measures of asepsis and on oral antibioprophylaxy with a fluoroquinolone during the whole period of treatment by leeches.

## Introduction

Leeches have been used for therapeutic purposes since 3500 B.C. Their qualities were discovered in former Egypt, China and India. A mural painting representing a nurse applying a leech to a patient’s forehead was discovered in a grave in Thebes (origin of the pharaohs of the 18th dynasty). The first written reference goes back to the second century B.C. and deals with the treatment of poisonous bites. The use of leeches reached a peak in the nineteenth century for various indications: laryngitis, ophthalmic problems, cerebral apoplexy, obesity, and mental disorders. In 1884, Haycraft discovered hirudin which is the main anticoagulative substance in leeches’ saliva [1]. In 1955, Markwardt was the first to isolate hirudin from the pharyngeal glands of leeches. Nowadays, this substance is only produced by genetic engineering. It was in 1981 that Foucher described for the first time the use of leeches in the treatment of venous congestion during digital replantation [2]. Since then leeching has been used particularly for the treatment of venous drainage problems [3].

Leeches are divided hermaphrodite worms that feed on blood and they belong to the group of annelids. Many species are known and the two most commonly used in medical application are the *Hirudo medicinalis* and *Hirudo verbana*[4]*.* Their length is about 12cm and their weight is 2g to 5g. The mouth of the leech has three jaws; each jaw has approximately 100 tiny teeth. A leech also has a posterior suction device that it uses for stability. Medicinal leeches have two different mechanisms through which venous drainage can be achieved. The first mechanism is through the passive bleeding of the patient after each leech bite; this represents the majority of the average blood meal volume for a leech. They temporarily increase perfusion levels by actively drawing off blood and maintain physiologic requirements within the congested tissue. Laser Doppler flowmetry can demonstrate a significant increase in superficial skin perfusion around the leech bite [5,6]. Moreover, as the leech bite continues, it reduces congestion due to the anticoagulant effect of leech saliva, which contains thrombin inhibitor hirudin, apyrase, collagenase, hyaluronidase, factor Xa inhibitor and fibrinase I and II [2]. A leech consumes 5mL to 15mL of blood and induces oozing on the site of attachment of between 50mL and 100mL of blood during the 24-hour to 48-hour period after the leech is detached.

**Figure 1:**
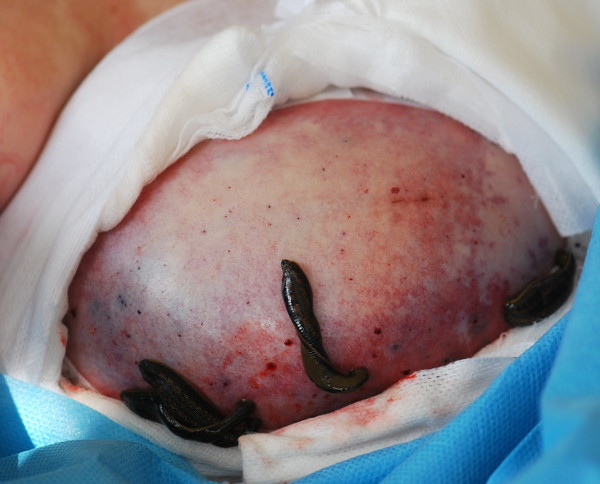
Venous congestion treated by leeches.

In clinical practice, the area that needs to be treated has to be cleaned with an antiseptic solution then with physiological serum because the leeches are very sensitive to odors [7]. During the process, leeches are handled with gloves. If the leech does not stick to the right place, then the leech has to be moved to the right area. When the leeches have ingested enough blood, they will fall away by themselves. Removing the leeches by hand can cause small phlegmons which are sources of infection. Leeches are applied once or twice a day until capillary circulation is restored (4 or 5 days; Figure 1).

In France, medicinal leeches are supplied by Ricarimpex®. Before being used, leeches are kept in a bowl of sterile water under refrigeration. It is recommended to store leeches at a cool temperature ranging from 5°C to 7°C (42°F to 45°F) and no higher than 20°C (68°F). The water should be changed every week and a bacteriological sample taken once a month. Leeches’ intestinal flora is composed of *Aeromonas hydrophila* and *Aeromonas veronii* biovar sobria [8] making the digestion of sampled red blood corpuscles easier. Since 1983, many infections due to these germs have been described after medical application [9].

We report two cases of septicemia related to *A. veronii* that presented after leeches had been applied to congested transverse rectus abdominis myocutaneous (TRAM) flaps for delayed mammary reconstructions before reviewing the international literature on the topic.

## Case presentation

### Patient number 1

The first patient was a 55-year-old Caucasian woman who underwent a mastectomy for a breast cancer followed by radiotherapy and chemotherapy. Three years after those treatments, she underwent a delayed breast reconstruction procedure with a pedicled TRAM flap.

Due to a massive and immediate venous congestion, she had to undergo 12 hours later a second operation and the flap had to be moved back to the initial position. Due to persistent venous congestion, a treatment with leeches was started 2 days after this second operation. Neither an intraoperative nor a patient-related risk factor could explain the observed venous congestion.

On the sixth postoperative day, following 4 days of leeching, the vascular status of the flap improved, but the patient showed a clinical presentation of septicemia associated with 39.2°C hyperthermia, a white blood cell count of 27,44 10^9^/L and a C-reactive protein (CRP) level increased by 153mg/L.

While blood cultures and local bacteriological swabs were being analyzed, an intravenous antibiotic therapy was started including vancomycin (1.5g/day) and cefotaxime (6g/day) for 10 days, and amikacin (350mg/day) for 2 days.

*A. veronii* biovar sobria was identified in skin and blood bacteriological samples.

The fever ceased after 4 days of antibiotic treatment. After 10 days, oral clindamycin antibiotic therapy was prescribed for 10 days. The flap was moved back to the mammary position at day 12 after 48 hours of apyrexia. The patient left the hospital at day 19.

### Patient number 2

The second patient was a 56-year-old Caucasian woman who underwent a mastectomy for a relapse after a first breast cancer treated by tumorectomy followed by radiotherapy. Despite the possibilities of immediate breast reconstruction, she refused to undergo such a procedure. Ten months later, she was motivated for a delayed procedure. Because we were not used to performing deep inferior epigastric perforator flaps at that time, she had a breast reconstruction using a pedicled TRAM flap.

Despite the absence of intraoperative complications and patient-related risk factors, the flap presented an early venous congestion that was treated from the second to the fifth postoperative day by leeching and heparin-moist gauzes. A partial necrosis settled in the mammary and abdominal area.

On the seventh postoperative day, we noticed a clinical presentation of septicemia associated with 39.8°C hyperthermia, a white blood cell count of 15 × 10^9^/L and a CRP level increased by 114mg/L.

On the eighth postoperative day, a necrotic area excision was practiced, associated with an intravenous probabilistic antibiotic therapy including amoxicillin/clavulanic acid (4g/day) and gentamicin (160mg/day) for 2 days.

*A. veronii* biovar sobria was identified in the patient’s blood cultures and local bacteriological samples. An antibiogram showed resistance to amoxicillin/clavulanic acid, which was stopped at day 10 and replaced by cefotaxime (6g/day) for 10 days followed by oral ofloxacin (400mg/day) for eight days. The fever ceased on the eleventh postoperative day after four days of antibiotic treatment. She left the hospital at day 20.

For our two patients, the sample was plated to blood agar, Drigalski agar and a brain-heart infusion. The isolate was identified by using a Vitek 2 Gram-negative card (bioMérieux Inc., Marcy L’étoile, France). Antimicrobial susceptibility was tested by using a 24-hour disk-diffusion method on Mueller-Hinton agar plates incubated at 37°C in an ambient air incubator.

## Discussion

Similar cases of infections due to leeching on TRAM flaps have been described in the literature [10]. It was in 1983 that Whitlock *et al*. suggested for the first time that there could be a risk of infection by *A. hydrophila* attached to leeching. Whitlock showed the presence of *A. hydrophila* on the leech’s body by making bacteriological samples. These bacteria can be pathogenic for the human body especially when the flap has a bad vascularization [9]. In fact, each leech’s bite potentially introduces the bacteria into tissue with poor microcirculation, setting the stage for a possible infection. In the literature, many cases of infection after leeching have been described [11]: 18 cases in 1992 by Lineaweaver [12], 19 cases in 1996 by De Chalain [13], 7 cases in 2002 by Sartor [14], and 47 cases in 2007 by Bauters [15]. The rate of infection can be important varying from 2.4% to 20.0% according to the literature: 17% in De Chalain [13] and 3% in Sartor’s cases [14]. De Chalain *et al.*[13] described 19 cases of leeche-related *Aeromonas* infection. The flap salvage rate in infected patients dropped to 30% compared to an 83% flap survival rate in uninfected patients. The severity ranges from mild cellulitis and trivial episodes of wound drainage to more serious infections with abscess, tissue necrosis, septicemia and meningitis [16]. Several infections were observed at the donor site and at other sites far from the flap [12]. Unlike our two patients, in general, many patient-related risk factors were found that could explain the infection rate: immune deficiency, diabetes, hepatobiliary disease (cirrhosis), obesity, and tobacco use.

Infection starts most of the time during the first 10 days, but in some cases infection can appear a few weeks after leeching [16]. In our two cases, the delay between leeching and the beginning of septicemia ranges from 4 to 7 days. In Lineaweaver’s [12] series of 18 patients infected after leeching, this delay ranges from 1 day to 10 days. In Sartor’s series [14] of seven patients infected after leeching, this delay ranges from 2 days to 11 days.

Numerous germs have been identified from leeches [17]. The saliva from *H. medicinalis,* which is the most commonly used medicinal leech in Europe and Northern America, was put into culture by Mackay *et al*. and they showed the presence of *A. hydrophila* and *Aeromonas sobria*[18]. The most common germ is *A. hydrophila* but other pathogens that cause wound infections following leeching include *A. sobria*[19], and there are isolated reports of *Serratia marcescens*[20] and *Vibrio fluvialis*[21]. *A. veronii* biovar sobria was found in blood cultures as well as in local bacteriological samples of our two patients.

The *Aeromonas* produces a β-lactamase that induces a resistance to penicillin and first-generation cephalosporins [22]. This resistance is confirmed on the antibiogram of our two patients (Table 1). The antibiotic therapy using amoxicillin/clavulanic acid prescribed to the second patient was not efficient. Yet, as mentioned by Hermansdorfer [23], the third-generation cephalosporins, ciprofloxacin and aminosids are efficient on *Aeromonas*. Our two patients did not receive the same antibiotics because they were treated in two different hospitals of the same area. For the first patient the antibiotic therapy was quickly effective whereas for the second patient the antibiotic therapy became active only after associating cefotaxime to aminosids. The symptoms of our two patients disappeared after 4 days of antibiotherapy.

**Table 1:** Patients’ antibiogram

**Antibiotic**	**Patient 1**	**Patient 2**
Amoxicillin	R	R
Amoxicillin/clavulanic acid	R	R
Piperacillin	S	S
Tazocillin	S	S
Cefalotin	R	S
Cefotetan	S	
Cefotaxime	S	S
Ceftazidim	S	S
Cefepim	S	
Imipenem	S	S
Aztreonam	S	S
Gentamicin	S	S
Tobramycin	S	S
Amikacin	S	S
Isepamicin	S	
Ofloxacin	S	S
Ciprofloxacin	S	S
Trimethoprim/Sulfamethoxazole	S	S

R: Resistant, S: Sensitive.

Before use and in order to avoid human infection, some authors propose immersing leeches in a solution of antibiotics for a few days or in a solution of chlorhexidine at 1:5000 before therapeutic use. Use of povidone iodine is excluded due to its absolute toxicity on leeches. Hokelek *et al*. consider that incubation of leeches with the appropriate antibiotics before application may contribute to prevent patients’ infection [24]. Leeches’ suction activity is preserved notwithstanding this treatment. Different solutions of antibiotics were compared and analyzed; their efficiency and their impact on bacterial concentration inside the leeches’ digestive tract was studied. The results showed that optimum eradication of bacteria from leeches was obtained with a dosage of 500μg per mL of ciprofloxacin and 1000μg per mL of ceftriaxone. Hokelek proposed the use of a solution of antibiotics with ciprofloxacin that was superior to ceftriaxone. Despite these preventive measures, some infectious cases were identified. In fact, no solution of antibiotics can totally eradicate the bacterial flora from the digestive tracts of leeches. Lineaweaver [25] nevertheless noticed an important decrease in the concentration of bacteria inside the digestive tracts of leeches when the patient is under an effective antibiotic therapy. Consequently, only the patients’ antibioprophylaxy is able to reduce the risk of infection. In 2002, Chepeha *et al*. [26] used double coverage with ciprofloxacin and trimethoprim/sulfamethoxazole prophylaxis in eight patients treated for venous congestion with an average of 215 leeches per patient without any *Aeromonas* infection reported. In 2004, Whitaker *et al*. [27] proposed a prophylactic protocol for the patient based on an intravenous antibioprophylaxy associating a fluoroquinolone such as ciprofloxacin and an aminosid during the complete period of leeching. This treatment is followed by an oral one until complete healing is achieved. In 2007, Knobloch *et al*. [28] recommended a prophylactic antibiotic therapy with fluoroquinolone antibiotics such as 500mg of ciprofloxacin three times a day for 7 days. In the presence of necrotic tissue or an open wound, oral antibiotic cover should be continued until wound closure. Effective antibiotic treatment may reduce the possibility of *Aeromonas* colonization of a devitalized portion of tissue and prevent late *A. hydrophila* infection [29].

Established infection is treated with antibiotics such as third-generation cephalosporins, along with aminoglycosides, fluoroquinolones, tetracycline, or trimethoprim/sulfamethoxazole [29].

As for our two patients, Hermansdorfer’s and Braga’s studies reveal that the *Aeromonas* is sensitive to fluoroquinolone, particularly ofloxacin and ciprofloxacin [23,30]. Ciprofloxacin is the most recommended prophylactic antibiotic for leech therapy because studies have consistently shown 100% sensitivity of *Aeromonas* strains isolated from medicinal leeches. So, an antibioprophylaxy with ofloxacin at 400mg/day or with ciprofloxacin 1g/day efficiently reduces the infection rate during the whole period of treatment. Tissue penetration is good and side effects are limited. Ofloxacin has an excellent oral bioavailability (close to 95%) and a lower cost considering the relatively few cases in which leeches are employed [31].

In 2011, Wang *et al*. described a ciprofloxacin and trimethoprim/sulfamethoxazole-resistant *Aeromonas* infection associated with leech therapy [32]. Studies that examined the antibiotic resistance profile of *Aeromonas* strains in industrial fish farms found a resistance to ciprofloxacin and to trimethoprim/sulfamethoxazole estimated to be 3% and 1%, respectively [33,34]. Currently, ciprofloxacin and trimethoprim/sulfamethoxazole resistances in *Aeromonas* are rare. In order to determine appropriate antibiotic prophylaxis on local resistance patterns, surgeons and infectious disease specialists have to collaborate. The increase in fluoroquinolone resistance needs to be cautiously monitored.

## Conclusion

The rate of infection after leeching is not negligible. It varies from 2.4% to 20.0% according to the literature [11,12]. The concentration of Aeromonas inside the digestive tracts of leeches largely decreases when the patient is under antibiotic therapy. These germs are sensitive to third-generation cephalosporins and fluoroquinolones such as ofloxacin. We recommend preventive treatment based on classical measures of asepsis and on oral antibioprophylaxy with fluoroquinolones such as ofloxacin at 200mg twice a day during the whole period of treatment by leeches.

## Consent

Written informed consent was obtained from the two patients for publication of these case reports and any accompanying images. A copy of the written consent is available for review by the Editor-in-Chief of this journal.

## Competing interests

The authors declare that they have no competing interests.

## Authors’ contributions

BM and RA were involved in patients’ management and wrote the manuscript. CBR also reviewed the literature. CBR operated on the first patient and JBA on the second one. All authors read and approved the final manuscript.
